# Supplementary figures and images for: Fine-tuning spatial-temporal dynamics and surface receptor expression support plasma cell-intrinsic longevity
Source: eLife. 2024 Jun 18;12:RP89712. doi: 10.7554/eLife.89712 (PMC11186632; doi:10.7554/eLife.89712)

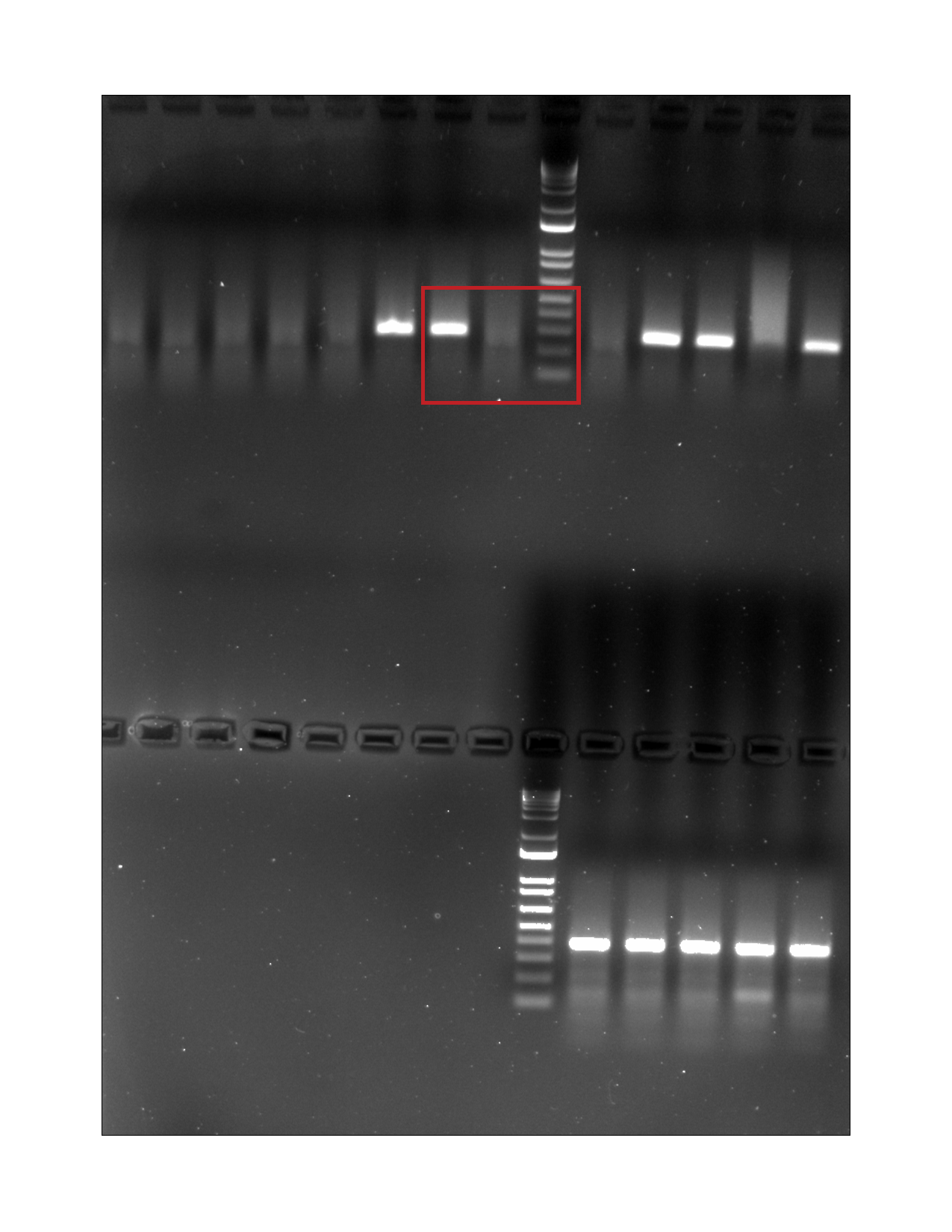

Supplement: Figure 1—figure supplement 1—source data 1. [file elife-89712-fig1-figsupp1-data1.tiff]
